# Stunting and soil-transmitted-helminth infections among school-age pupils in rural areas of southern China

**DOI:** 10.1186/1756-3305-3-97

**Published:** 2010-10-13

**Authors:** Yu Shang, Lin-Hua Tang, Shui-Sen Zhou, Ying-Dan Chen, Yi-Chao Yang, Shao-Xiong Lin

**Affiliations:** 1 National Institute of Parasitic Diseases, Chinese Center for Disease Control and Prevention (China CDC), 207 Rui Jin Er Road,Shanghai 200025, PR China; 2 Hebei Provincial Center for Woman and Child Health Care, Shijiazhuang 050031, China; 3 Guangxi Autonomous Regional Center for Disease Control and Prevention, Nanning 530000, China; 4 Hainan Provincial Center for Disease Control and Prevention, Hankou 570000, China

## Abstract

**Background:**

Stunting and soil-transmitted helminth (STH) infections including ascariasis, trichuriasis and hookworm remain major public health problems in school-age pupils in developing countries. The objectives of this study were to determine the prevalence of stunting for children and its association with three major soil-transmitted helminths (STH) in rural areas of southern China. The study also aims to determine risk factors for stunting and to provide guidance on the prevention and control of stunting and STH infections for future studies in this field.

**Results:**

A cross-sectional survey was carried out in the poor rural areas in Guangxi Autonomous Regional and Hainan Province where STH prevalence was higher between September and November 2009. Pupils were from 15 primary schools. All the school-age pupils aged between 9 and 12 years old (mean age 11.2 ± 3.2 years), from grades three to six took part in this study. Study contents include questionnaire surveys, physical examination and laboratory methods (stool checking for eggs of three major STH infections and haemoglobin determination was performed for the anaemia test). Finally 1031 school-age pupils took part in survey. The results showed that the overall prevalence of stunting (HAZ < 2SD) was 25.6%, based on the WHO Child Growth Standards (2007). Risk factors for stunting based on logistic regression analyses were: (1) STH moderate-to-heavy intensity infections (OR = 1.93;95%CI:1.19,3.11); (2) anaemia (OR = 3.26;95%CI: 2.02,5.27); (3) education level of mother (OR = 2.13; 95%CI: 1.39,3.25). The overall prevalence of major STH infections was 36.7%, STH moderate-to-heavy intensity infections was 16.7%. The overall prevalence of ascariasis, trichuriasis, hookworm and co-infection were 18.5%, 11.2%, 14.7% and 9.1% respectively. The prevalence of anaemic children (HB < 12 g/dl) was 13.1%.

**Conclusion:**

The present study showed that stunting was highly prevalent among the study population and STH infection is one of the important risk factors for stunting, with moderate-to-heavy intensity infections being the main predictor of stunting. Hence, additional interventions measures such as to promote de-worming treatment, to enhance health education and to improve hygiene and sanitation in order to reduce stunting in this population, are needed throughout the primary school age group.

## Background

Although great development in socio-economic status has occurred in recent years, malnutrition and intestinal parasitic infections are still common public health problem in school-age pupils in many parts of the world, particularly in developing countries. In endemic areas, school-age pupils suffered from the greatest burden of infections with three major soil-transmitted helminths (STH), which threaten their physical development. It has shown that the prevalence of malnutrition is still high in rural areas, despite implementation of programs for control and prevention around the world[1,2]. Indicators of malnutrition include wasting, stunting and being underweight[3]. Stunting or low height-for-age (HAZ), is thought to be a good indicator of malnutrition and represents a status of chronic nutritional stress[4]. It can lead to both short-and long-term adverse events in children, including effects on health, growth, cognition and educational outcomes[5-7]. STH infections are also chronic diseases, but their detection is not easy because of the absence of clinical symptoms. Stunting and three major soil-transmitted helminth (STH) infections can both bring a major burden of disease for children. In addition, stunting happening in children can last to adulthood, and for woman, it can lead to low birth weight babies who are likely to develop stunting.

Some studies in many countries has proved that parasitic infections, especially *Ascaris*, *Ttrichuris *and co-infections were associated with stunting in school-age pupils and that de-worming treatment can improve children's nutritional status[8,9]. In China, although the nutritional status of children has improved very much recently, the prevalence of stunting and STH infections is still high. Thus stunting and STH infections remains an important matter that affects Chinese children, but there are few related research data in the literature.

The objectives of this study were to determine the prevalence of stunting using the new WHO Child Growth Standards and its association with three major soil-transmitted helminth (STH) infections in poor rural areas of southern, China. The study also aims to determine risk factors for children's stunting and to provide guidance on the prevention and control of stunting and STH infections for future studies in this field.

## Materials and methods

### Study areas and study population

The cross-sectional survey was carried out in two poor rural areas in Guangxi Autonomous Regional Rongshui County and Hainan Province Dingan County, China, from September to November 2009, Pupils were from 15 primary schools. All the school-age pupils aged between 9 and 12 years old (mean age 11.2 ± 3.2 years), from grades three to six took part in this study. Studies conducted in both areas showed high prevalence rates of three major STH infections [10]. In the two areas, school-based de-worming treatment had never been used. Of the 15 schools, 10 are located in Dingan County, a champaign area with geographical coordinate 19°40'00 N latitude and 110°25'00 E longitude and altitude 210 m above sea level. 5 schools are located in Rongshui County in a mountainous region, with geographical coordinate 24°33'00 N latitude and 109°4'00 E longitude and altitude 243 m above sea level.

### Questionnaire surveys

Questionnaires for collecting information were designed by ourselves and contained detailed information on personal, parental and socio-economic status. Questions included age, sex, education level and occupation of parents and household income, etc.

### Physical examination

Body weight and height were measured using the standardized procedures mentioned in [11]. Weights of the pupils were recorded using a scale to the nearest 0.1 kilograms (kg). Heights were measured to 0.1 centimeters (cm). Pupils wore minimum clothing and no shoes. Age was calculated from the date of birth in school records to the date of visit.

### Laboratory methods

Stool samples were examined by the Kato-Katz technique (thick smear 41.7 mg). Containers for collection of stools were dispensed to each class and labeled separately for pupils, Pupils were asked to collect and deliver samples of their faeces to school the next day.

Blood samples (4 ml) were collected by venipuncture, which were then immediately put into a tube containing 5 ml anticoagulant and used to measure haemoglobin concentration.

All stool samples and blood samples examinations were completed by the staff of the National Institute of Parasitic Diseases, Chinese Center for Disease Control and Prevention and the staff from the local Center for Disease Control and Prevention.

### Data management and analysis

Anthropometric indices were calculated using the new World Health Organization Child Growth Standards (WHO AnthroPlus, 2007) [12]. Height-for-age Z-score (HAZ) was used as an indicator for stunting. Stunting was defined as Z-score below -2 standard deviation (SD).

STH infections were expressed as the number of eggs per gram stool (EPG). Three Kato-Katz slides were prepared from each stool sample. Infection intensity was defined by number of eggs/gram (EPG) of faeces using the World Health Organization criteria for light-, moderate- or heavy-intensity infection for ascariasis, trichuriasis and hookworm eggs. For *T. trichiura*, the light intensity infection category was defined as 1-999 EPG and the moderate to heavy intensity infection category was defined as ≥1000 EPG. For ascariasis, light-and moderate to heavy intensity infection categories were defined as 1-4999 EPG and ≥5000 EPG, respectively. For hookworm, light-and moderate to heavy intensity infection categories were defined as 1-1999 EPG and ≥2000 EPG, respectively.

The diagnostic criteria for anaemia was defined as a haemoglobin concentration less than 12 g/L [13].

### Statistical analysis

EpiData3.0 was used to establish a database with the data collected. Statistical analysis of the data was performed using the statistical package for Social Sciences for Windows SPSS (version 16.0). Z-score was obtained by WHO AnthroPlus software 2007. This software is for the global application of the WHO reference for 5-19 years to monitor the growth of school-age children and adolescents. Analysis of co-variance (ANCOVA) models were constructed using HAZ. Chi-squared test was used to examine differences for proportions. Odds ratios (OR) calculated by logistic regression were presented to determine risk factors for stunting.

### Ethical considerations

This study was approved by the ethics committees of the National Institute of Parasitic Diseases, Chinese Center for Disease Control and Prevention (China CDC). Questionnaire surveys, physical examination and laboratory work were conducted after the purpose of the study had been explained to participants, who were given the right to withdraw from the study at any time, without consequences. Written informed consent was obtained from each pupil's representative.

## Results

### Demographic characteristics of the pupils

**Figure 1:**
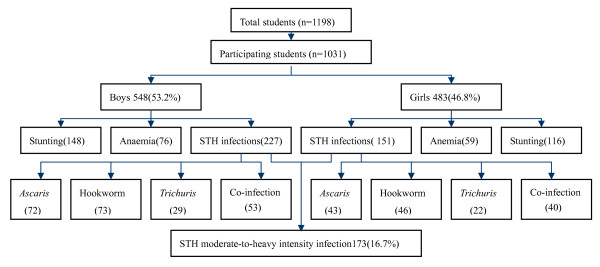
**Results of cross-sectional survey in study areas of China**.

A total of 1198 pupils took part in the study. Among them, 108 pupils were unable to provide stool samples and another 59 pupils fail to fill out questionnaires. The final total population of pupils who were able to provide complete information (questionnaires, stool samples, blood samples, and physical examination) was 1031(participation rate = 86.1%), including boys 548 (53.2%) and girls 483 (46.8%) There was no significant difference between boys and girls (Fig. 1).

The general demographic characteristics of 1031 pupils were presented as follows: pupils showing stunting 264, prevalence of stunting was 25.6%. 378 pupils were infected with STH and 173 pupils had moderate-to-heavy intensity of infection. Prevalence of STH infections and moderate-to-heavy intensity infections were 36.7% and 16.7%, respectively. Ascaris, hookworm and *Trichuris *infections were 115 (11.2%), 121 (11.7%) and 51 (4.9%), respectively. Anaemia pupils were 135, prevalence of anaemia (HB < 12 g/dl) was 13.1%. No pupils had severe anaemia (HB < 7 g/dl). Among anaemic pupils, prevalence of stunting was 40.7%. Chi-squared test: *x*^*2 *^= 18.68, p = 0.00 (p < 0.05).

### HAZ of STH infections group compared with non-infected group

Mean HAZ of 173 STH moderate-to-heavy intensity infections was (-1.599 ± 1.3), and the mean HAZ of non-infected was (-1.292 ± 1.25). Chi-squared test: *x*^2 ^= -2.952, p = 0.003. The difference was statistically significant (p < 0.05). We could conclude that STH moderate-to-heavy intensity infections influenced children's physical development, especially height. Height reflects a chronic nutritional status.(Table 1)

**Table 1:** Comparision of Height-for-age Z-score among pupils in different groups ($\bar{\text{X}}$ ± SD)

	**moderate-to-heavy STH infection**^**a **^**(173)**	**light STH infection**^**b **^**(205)**	**control group**^**c **^**(653)**	F	P	comparison among groups
Boys (548)	100	127	321			
HAZ	-1.63 ± 0.82	-1.32 ± 1.07	-1.29 ± 0.92	5.18	0.006	a < b*, a < c*
Girls (483)	73	78	332			
HAZ	-1.58 ± 0.94	-1.49 ± 0.95	-1.25 ± 1.15	3.99	0.019	a < c*

### Univariate analysis for stunting

Table 2 shows the results of univariate analysis by chi-square test. Age, education level of mother, anaemia, *Ascaris *infection only, *Trichuris *infection only, moderate-to-heavy intensity infections influenced on stunting.

**Table 2:** Characteristics and risk factors for stunting univariate analysis

Characteristics	number of students(%)	**number of stunting**^**1 **^**(%)**	OR(95%CI)
Total	1031	264(25.6)	
Age			
9-	181 (17.6)	26(14.4)	1.00
10-	239 (23.2)	35(14.6)	1.02(0.59,1.77)
11-	285 (27.6)	58(20.4)	1.52(0.92,2.53)
12-	326 (31.6)	145(44.5)	4.78(2.99,7.64)
Sex			
Female	483 (46.8)	117(24.2)	1.00
Male	548 (53.2)	147(26.8)	1.15(0.87,1.52)
Education level of mother			
Junior high school and above 391 (37.9)		88(22.5)	1.00
Primary school and below	640 (62.1)	176(27.5)	1.31(0.97,1.75)
Anaemia^**2**^			
No	896 (86.9)	209(23.3)	1.00
Yes	135(13.1)	55(40.7)	2.26(1.55,3.29)
STH infections			
No	653 (63.3)	148(22.7)	1.00
Yes	378 (36.7)	116(30.7)	1.51(1.14,2.01)
Ascaris infection only			
Non-infection	653 (63.3)	148(22.7)	1.00
Yes	115(11.2)	46(40.0)	2.28(1.80,3.45)
Hookworm infection only			
Non-infection	653 (63.3)	148(22.7)	1.00
Yes	121 (11.7)	24(19.8)	0.84(0.52,1.37)
Trichuris infection only			
Non-infection	653 (63.3)	148(22.7)	1.00
Yes	51 (4.9)	23(45.1)	2.8(1.57,5.01)
Moderate-to-heavy infection			
Non-infection	653 (63.3)	148(22.7)	1.00
Yes	173 (16.7)	67(39.9)	2.16(1.50,3.08)
Co-infection			
Non-infection	653 (63.3)	148(22.7)	1.00
One infection	287 (27.8)	93(32.4)	2.74(1.97,3.80)
Two-Three infection	94 (9.1)	22 (23.4)	1.04(0.63,1.74)

^1 ^Stunting was defined as z-scores < -2 standard deviation.

^2 ^Anaemia was defined as a haemoglobin concentration <12 g/dl.

### Logistic regression risk factors for stunting

Table 3 shows that the dependent variable is stunting. The covariates that were finally retained in the model were STH moderate-to-heavy intensity infections, anaemia and educational level of the mother. Risk factors for stunting were (1) STH moderate-to-heavy intensity infections (OR = 1.93; 95%CI:1.19,3.11) (2) anaemia (OR = 3.26; 95%CI: 2.02,5.27) (3) education level of mother (OR = 2.13; 95%CI: 1.39,3.25).

**Table 3:** Logistic regression risk factors for stunting among pupils of 9-12 years

Risk factor	*P*-value	Odds ratio	95%CI
Stunting(n = 264)			
STH moderate to heavy intensity infections	0.007	1.93	1.19-3.11
Anaemia	0.000	3.26	2.02-5.27
Education level of mother	0.001	2.13	1.39-3.25

## Discussion

Children's physical development is affected by inheritance, nutrition, environment, exercise and living conditions, etc. Nutrition plays a particularly important role. With the rapid development of economic improvement and a changing natural environment, incidence of stunting and STH infections has also declined. Although the World Health Organization had estimated that an overall prevalence of stunting has fallen in developing countries from 47% in 1980 to 33% in 2000, stunting in school-age pupils is common in developing countries with the stunting prevalence being higher in primary schools. Malnutrition is still a major public health problem in poor areas in developing countries[14,15]. In these study areas, health knowledge and health status of pupils had improved considerably, but our results indicate in the rural areas, prevalence of stunting in school-age pupils was 26.5%. School-aged pupils had the highest prevalence and intensity of STH infections. Stunting represents a chronic state of nutritional stress. That is to say, these pupils had long history of lack of nutrition, or they were infected by some other disease.

To evaluate children's growth and development needs a comparable reference value. WHO recommends Z-score as the best method. It is calculated according to weight and height and reflects children's growth and development. It compares the distribution of Z-score of the tested group with that of the reference group, then to find the tested group's nutrition conditions. Z-score can evaluate children's general nutrition conditions. In this study, mean HAZ data were all negative, which illustrated the fact that the nutritional condition in the tested group had a significant difference compared with WHO standard. In addition, HAZ value also reflects the whole lower economic level and human body exposesed to poor conditions. In this study, STH moderate-to-heavy intensity infections influenced children's growth and development, and there is significant difference between its mean HAZ data and that of non-infection group (p < 0.05).

The present data showed that STH moderate-to-heavy intensity infections are important risk factors for stunting. STH infections are widely distributed throughout the tropics and subtropics. STH infections in people remains a worldwide public-health threat for as long as poverty persists in the developing world. The STH infections are one of the world's most important causes of physical and intellectual growth retardation[16]. Previous studies had found *Trichuris*, *Ascaris *or co-infection were all associated with stunting[8,17]. Soil-transmitted helminths lead to nutritional loss. Mechanisms by which STH infections can lead to stunting in pupils including decreased appetite and food intake, depletion and impaired absorption of micronutrients and anaemia. The blood loss which is associated with *Trichuris *infection can lead to chronic dysentery, iron deficiency, iron deficiency anaemia and poor growth rate[18]. Well-nourished children, who have better nutrient reserves, are thought to be less vulnerable to the adverse effects of parasitic infections. Previous studies have showed growth improvements of pre-school children after anthelmintic treatment[19,20]. Longitudinal studies have also demonstrated that stunted children continue to deviate from growth standards as they get older[21]. Our results suggest that the risk of stunting continues to grow as age increses. So, treatment of helminth infections in school-age children may improve growth in areas where stunting and helminth infections are prevalent.

In this study, anaemia was also found to be associated with stunting and was a risk factor for stunting. It is stated by WHO that anaemia remains one of the most intractable public health problems in Africa, contributing to a quarter of Africa's nutrition-related Disability Adjusted Life Years (DALYs) lost[22]. Anaemia in children can be caused by iron deficiency and by health factors such as parasitic infection[23]. In this study, 135 pupils were anaemic (HB < 12 g/dl) with a prevalence 13.1%. Among anaemic pupils, the prevalence of stunting was 40.7%. the difference was statistically significant (p < 0.05).

In this study, it is clear that mother's education level was an important risk factor for children's growth and development. Maternal education has been previously found to be an important risk factor for childhood malnutrition[24,25]. Thus, if mothers have a better education and knowledge on health care, they may have better knowledge of proper health and nutrition behaviours for their families. So, health and nutrition educational interventions targeted to mothers and pupils are needed.

## Conclusion

In conclusion, this study demonstrated that specific interventions efforts should be stepped- up for preventing stunting in school pupils. Firstly, in school, group de-worming treatment needs to be developed, and the prevention and control of STH infections should be part of integrated school-based health program. Secondly, health and hygiene knowledge education need to be provided for parents, especially for mothers.

## List of abbreviations

STH: soil-transmitted helminth; WHO: World Health Organization; HAZ: Height-for-age Z-score; SD: standard deviation; EPG: eggs per gram; SPSS: Social Sciences for Windows; OR: Odds ratios; CDC: Centers for Disease Control.

## Competing interests

The authors declare that they have no competing interests, professional or personal competing interests related to this article. The funding agencies played no role in the design or implementation of the study, analysis or interpretation of the data, or the preparation and submission of the manuscript.

## Authors' contributions

TLH and SY conceived the study, organized the survey, coordinated and supervised the field work and the data entry and analysis. ZSS and CYD imputed data and assisted in the interpretation of the resulted. YYC and LSX provided technical support for data collection. SY drafted the manuscript and TLH revised the manuscript. All authors read and approved the final manuscript.
